# Clay Composites for Thermal Energy Storage: A Review

**DOI:** 10.3390/molecules25071504

**Published:** 2020-03-26

**Authors:** Denis V. Voronin, Evgenii Ivanov, Pavel Gushchin, Rawil Fakhrullin, Vladimir Vinokurov

**Affiliations:** 1 Department of Physical and Colloid Chemistry, Gubkin University, 119991 Moscow, Russia; denis.v.voronin@gmail.com (D.V.V.); ivanov166@list.ru (E.I.); guschin.p@mail.ru (P.G.); vinok_ac@mail.ru (V.V.); 2 Remote Controlled Theranostic Systems Lab, Educational and Research Institute of Nanostructures and Biosystems, Saratov State University, 410012 Saratov, Russia; 3 Bionanotechnology Lab, Institute of Fundamental Medicine and Biology, Kazan Federal University, 420008 Kazan, Russia

**Keywords:** phase change materials, montmorillonite, sepiolite, kaolinite, halloysite, diatomite, latent heat storage, paraffins, salt hydrates, composites

## Abstract

The development of novel materials and approaches for effective energy consumption and the employment of renewable energy sources is one of the current trends in modern material science. With this respect, the number of researches is focused on the effective harvesting and storage of solar energy for various applications. Phase change materials (PCMs) are known to be able to store thermal energy of the sunlight due to adsorption and release of latent heat through reversible phase transitions. Therefore, PCMs are promising as functional additives to construction materials and paints for advanced thermoregulation in building and industry. However, bare PCMs have limited practical applications. Organic PCMs like paraffins suffer from material leakage when undergoing in a liquid state while inorganic ones like salt hydrates lack long-term stability after multiple phase transitions. To avoid this, the loading of PCMs in porous matrices are intensively studied along with the thermal properties of the resulted composites. The loading of PCMs in microcontainers of natural porous or layered clay materials appears as a simple and cost-effective method of encapsulation significantly improving the shape and cyclic stability of PCMs. Additionally, the inclusion of functional clay containers into construction materials allows for improving their mechanical and flame-retardant properties. This article summarizes the recent progress in the preparation of composites based on PCM-loaded clay microcontainers along with their future perspectives as functional additives in thermo-regulating materials.

## 1. Introduction

The sustainable development of society is closely associated with the rational use of natural resources, including those for energy production. Currently, most of the produced energy originates from fossil fuels [1]. However, oil, natural gas, and coal are limited and non-renewable energy sources. Additionally, the growing consumption of fossil fuels makes a prominent environmental impact. The consumption of fossil energy resources may be reduced employing alternative energy sources such as solar energy. Solar energy is one of the most promising renewable energy sources due to its abundance, which, however, is not constant [2]. Therefore, the technologies for storing and transport thermal energy will facilitate the consumption of solar energy and reduce the consumption of non-renewable energetic resources [3,4]. For instance, this can be achieved by responsive energy management, which implies the solar energy accumulates when it is accessible and released when it is demanded [5].

Phase change materials (PCMs) can store and release thermal energy as latent heat of reversible phase transitions. Since the 1970s several attempts were made to incorporate PCMs into conventional building materials like wallboards to enhance the thermal comfort and reduce the energy required for heating or cooling of building interiors [6,7]. The first approach was a simple immersion of the wallboards in a molten PCM [8]. However, the incorporated PCMs lost their shape stability when turned to the liquid form and leaked to the surface. Therefore, it was concluded that PCMs have to be shape-stabilized first before the incorporation into construction materials. Various approaches were proposed, including encapsulation and impregnation methods. The encapsulation approaches vary from macroencapsulation to microencapsulation [9,10]. The employment of micron-sized PCM containers as thermo-regulating additives seems preferable as it significantly increases the surface area and, therefore, the heat transfer [11]. Alternatively, an impregnation in porous support may be employed [12,13], which is preferable for the inclusion of PCM into natural containers like clays and zeolites, instead of the preparation of artificial microcapsules.

Clay mineral microparticles are natural containers suitable for PCM encapsulation due to their intrinsic properties. Clay microparticles possess a porous structure, prominent specific surface area, and good absorbability [14]. The PCM mainly interact with clay minerals through capillary force, surface tension, hydrogen bond, and Van der Waals’ force that prevent PCM leakage inside the containers [15]. Clay minerals are abundant and their price is quite acceptable. Additionally, clay is a conventional component of many construction materials and, thus, the PCM/clay composites can be easily incorporated in their compositions. Clay microcontainers have good mechanical and thermal stability and compatible with various treatment procedures such as mixing, extrusion, molding, etc.

The preparation of PCM/clay materials has been studied widely. This review summarizes the data on the various types of PCM materials typically used for thermal energy storage, the general types of clay microcontainers with the most common examples, the approaches used for the composite’s preparation and the thermal properties of the PCM/clay composites concerning their latent heat storage capacity, thermal conductivity, and reliability. At the end of the review, the research trends with the future perspectives of PCM/clay composites are highlighted.

## 2. Thermal Energy Storage with PCM

**Figure 1:**
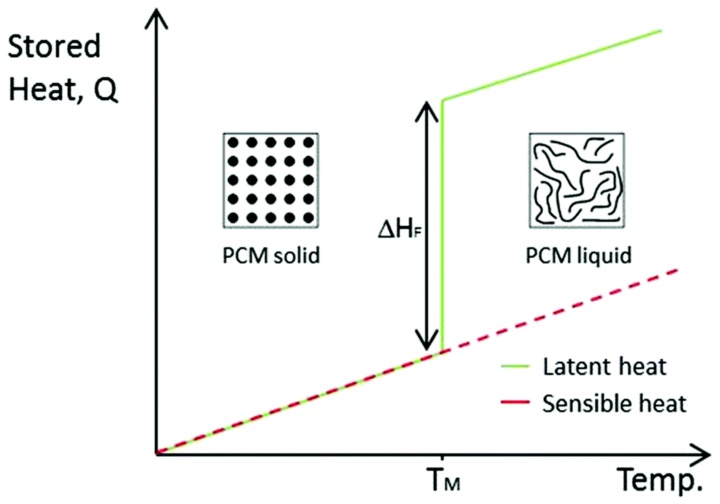
Comparison between SHS and LHS, ΔH_F_ is the latent heat of fusion during melting. T_M_ is the melting temperature. Reproduced with permission from [20]. Copyright The Royal Society of Chemistry.

Generally, there are three main approaches for thermal energy storage, which are sensible heat storage (SHS), latent heat storage (LHS) and thermochemical heat storage. Sensible heat implies the heat that can be directly measured and linearly related to the temperature fluctuations of the storage medium [16]. The ability to store sensible heat is defined by the heat capacity of the material. The typical example is water in district heating [17]. Thermochemical heat storage can be performed in the systems converting heat to the chemical energy and vice versa [18]. Finally, latent heat storage is associated with an enthalpy of phase transitions. Unlike the sensible heat, the term “latent heat” implies the material temperature remains the same during the phase transition. SHS and LHS approaches (Figure 1) have been extensively developed and employed for thermal energy storage [19]. However, SHS has lower energy storage density comparing to LHS and tend to lose thermal energy at any temperature. In turn, LHS has greater storage density within a much smaller temperature interval.

To be suitable for LHS, the material should undergo a phase transition in a given temperature range. This may include solid-liquid transition like freezing or melting, a liquid-gas transition like evaporation or condensation, or solid-solid transition attended with the changes in the crystalline structure of the material. Eventually, the efficiency of energy storage depends on the number of broken bonds [20].

**Figure 2:**
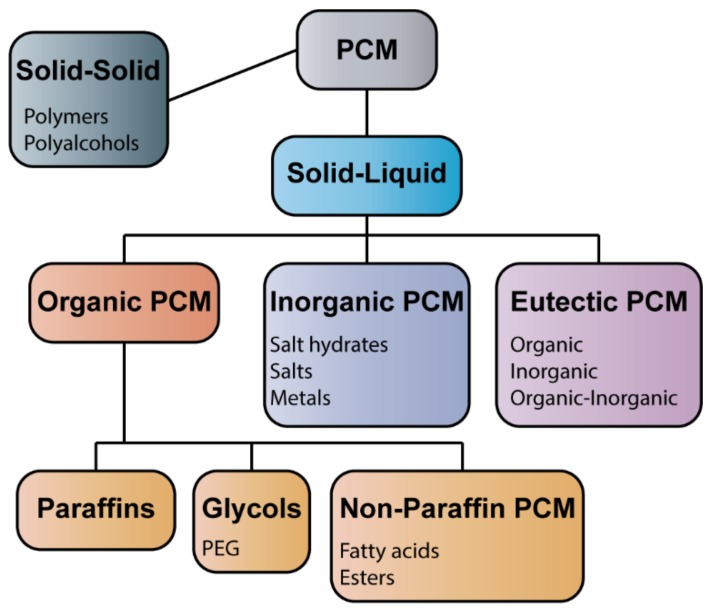
Classification of phase change materials (PCM) for latent heat storage applications.

From this viewpoint, the solid-gas transition (e.g., sublimation) appears the most efficient for thermal energy release yet is hardly applicable due to the large volume change. Therefore, the solid-liquid and solid-solid phase change materials (PCM) have been the most studied for potential practical applications (Figure 2). Although there is a number of recent works devoted to the development of solid-solid PCM (for instance, [21,22,23]), these materials have an intrinsically low heat of fusion. In turn, solid-liquid PCM combine high heat of fusion with the possibility of controllable volume change upon phase transition and attract much attention for thermal energy storage applications.

PCMs with the solid-liquid phase transitions can be divided into three main types: organic PCMs, inorganic PCMs, and PCM eutectics (Figure 2). Organic PCMs include paraffins, glycols and non-paraffin ones. Paraffin waxes are one of the most important types of PCM, generally consisting of mixtures of strait-chain *n*-alkanes containing 8 to 40 carbon atoms with the common formula (CH_3_-(CH_2_)_n_-CH_3_) [24]. A large amount of latent heat can be stored and released due to the melting and crystallization of (CH_2_)_n_ chains [25]. The advantages of paraffin waxes are chemical inert, recyclability of LHS, good stability below 500 °C, non-corrosive, little volume extension during melting. The shortages are low thermal conductivity, flammability, and high cost. Additionally, paraffin waxes are non-renewable as they are prepared from fossil fuel, while the commercially available paraffin waxes may contain volatile and carcinogenic substances [26]. 

Polyethylene glycol (PEG) is an attractive type of organic PCM due to its high enthalpy, wide transition temperature range, thermal and chemical stability, ease of chemical modification, low vapor pressure, competitive price possessing non-toxic and non-corrosive nature [27]. Importantly, LHS capacity and phase transition temperature of PEG are varied with the molecular weight. The disadvantages of PEG are low thermal conductivity and phase instability in the melting state.

The non-paraffin organic PCMs include fatty acids and other non-paraffin PCMs like esters. (CH_3_(CH_2_)_2n_COOH) fatty acids have high LHS capacity, limited supercooling, high stability in the proper temperature range, and can be produced from renewable sources. 

The inorganic PCMs include salt hydrates, salts, and some metals [28]. The salt hydrates are the major class of inorganic PCM. Generally, salt hydrates can be considered as a salt containing a specific number of water molecules within its crystal structure and described by the formula AB∙*n*H_2_O, where AB is some inorganic salt. The heating of a crystalline salt hydrate leads to the liberation of water molecules from the hydrate crystal and the formation of an aqueous salt solution. The process of solid-liquid transition in salt hydrates called dehydration, which is formally is not a real melting, however, is very close thermodynamically to the melting of the pure anhydrous salt [29]. Compared to organic PCMs, salt hydrates possess high volumetric LHS capacity, relatively high thermal conductivity, a wide range of melting temperatures along with non-toxicity, nonflammability, wide commercial availability and low cost. These make salt hydrates very attractive PCMs for mass application in building and industrial energy storage. However, there are some disadvantages as well. The main problem with salt hydrates is an incongruent melting [30,31]. The incongruent melting occurs when the anhydrous salt contained in the salt hydrate is partly soluble in water at the salt hydrate melting temperature. This results in the formation of two phases that are a saturated salt solution and anhydrous salt or lower salt hydrate precipitating at the bottom of the melt due to phase separation. The phase separation reduces the latent heat of fusion at the desired melting temperature and makes the hydrate more and more unstable with every further melting/solidification cycle. The supercooling is another common problem of salt hydrates [32]. The supercooling is an intrinsic property of the materials that is caused by a kinetic barrier of crystallization and basically can be calculated as the difference between melting and freezing temperatures at a given heating/cooling rate. The supercooling conditions depend on many factors affecting the supercooling rate. As a result, the material has to be cooled far beyond its freezing point to freeze that affects the storage density and thermal performance of salt hydrates.

Therefore, both organic and inorganic PCMs each have their advantages and disadvantages and a particular type of PCM should be chosen to meet the demands of a particular application. On the one hand, organic PCMs have better cyclic stability and are less prone to supercooling, but have a lack of shape stability leading to material leakage, inflammable and expensive. On the other hand, inorganic PCMs are low-cost for mass application and have better thermal properties than organic PCMs, but are less stable due to the phase separation and are prone to supercooling. However, the biggest common issue with both organic and inorganic PCMs is their low thermal conductivity restricting effective heat exchange with the environment. 

The encapsulation of PCMs in micro- and nanocontainers was suggested as an approach to overcome these limitations [33,34]. In particular, PCM encapsulation may protect the material from contact with the environment, increase heat transfer area and heat conductivity, improve shape and cycle stability [35]. Generally, the encapsulation of a PCM implies the formation of an organic or inorganic shell around the PCM core with chemical, physico-chemical and physico-mechanical approaches [10]. The preparation of microcapsules with organic PCMs has been studied widely. Generally, the first step in the encapsulation of organic PCMs is a preparation of an oil-in-water emulsion [33] that requires additional control of stability during the storage and technological operations [36]. The encapsulation of inorganic PCMs is less studied as it appears more challenging due to their good water solubility, polarity and changeable water content affecting the heat storage. One of the recently purposed approaches for encapsulation of inorganic PCMs is inverse emulsion interfacial polymerization for the preparation of a PCM core with an organic (poly(ethyl-2-cyanoacrylate) [PECA]) [37,38] or inorganic (SiO_2_) shell [39]. However, the polymeric microcontainers have poor thermal conductivity and relatively low content of core PCM material due to the thick polymeric shell required to effectively prevent material leakage [40]. In contrast, inorganic shells possess better thermal conductivity along with good mechanical strength and chemical stability. From this point, clay microparticles with abundant reserves across the world and very low cost are very attractive as natural containers for PCM loading.

## 3. PCM/clay Composites for Thermal Energy Storage

**Figure 3:**
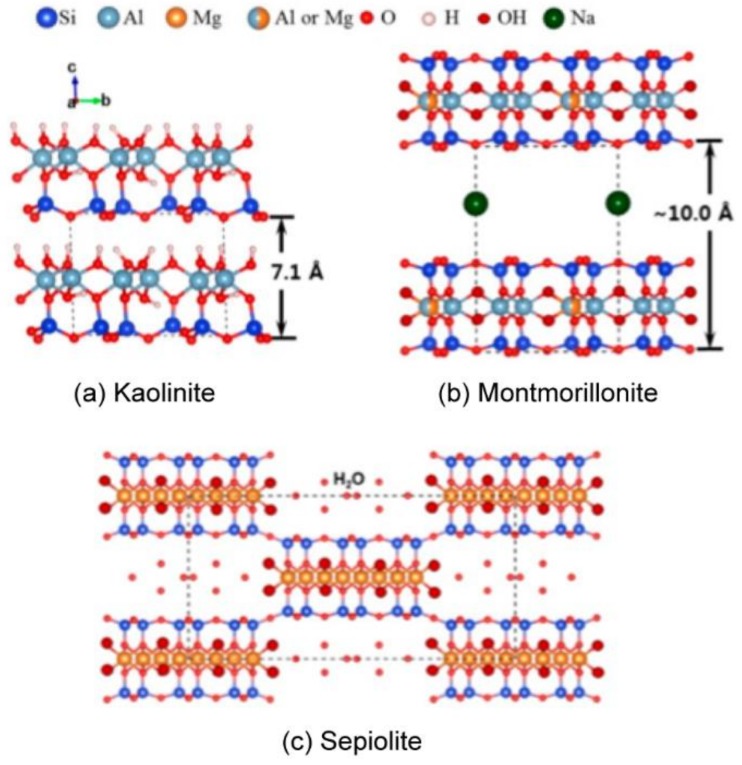
Crystal structure of (**a**) kaolinite, (**b**) montmorillonite, and (**c**) sepiolite, where the dashed lines represent the unit cell. Adapted with permission from [42]. Copyright Elsevier.

The clay mineral materials benefit from the porous structure along with a prominent surface area and intensively studied for PCM storing and supporting applications [14,15]. According to Gu et al. [41] depending on the internal layer structure the clay minerals can be divided into two types: amorphous and crystalline. In turn, crystalline clays can be subdivided into groups such as 1:1 type layered (e.g., kaolinite), 2:1 layered (e.g., montmorillonite), and 2:1 layered-chained (e.g., sepiolite) types (Figure 3).

The preparation methods of PCM/clay materials include vacuum impregnation, melting intercalation, melting adsorption and variations [43,44]. The main thermal properties of the PCM/clay composite materials are phase change temperature, the heat of fusion and freezing, supercooling temperature, thermal reliability and stability, and thermal conductivity. These properties are mainly determined by the loaded PCM and can be measured with the complex of thermal analysis measurements, including differential scanning calorimetry (DSC), thermogravimetric analysis (TGA), and several thermal conductivity methods (e.g., the Transient Plane Source (TPS) method) [45,46,47,48,49]. An important property of PCM/clay composites is the loading efficiency of PCM that is derived as the mass fraction of the PCM in the composite container. Loading efficiency can be figured out from the DSC analysis results as follows:

(1) $$LE=\frac{H_{composite}}{H_{PCM}}\times 100\%$$

where *H_composite_* is the enthalpy of the composite material and *H_PCM_* is the enthalpy of pure PCM. 

An another important parameter of PCM/clay composites that affects the latent heat storage and release efficiency is the crystallinity of the loaded PCM. The crystallinity of encapsulated PCMs may be lower than that of pure PCMs due to interface effects, which results in the formation of an amorphous PCM layer and therefore a lower LHS than expected [50,51]. The crystallinity (*F_c_*) of encapsulated PCM can be calculated from the DSC data as well using the expression:

(2) $$F_{c}=\frac{H_{composite}}{H_{PCM}\times LE}\times 100\%$$

where *H_composite_* and *H_PCM_* are enthalpy of the composite material and pure PCM, respectively, and LE is the loading efficiency of the PCM. 

### 3.1. Montmorillonite-Based PCM Composites

Montmorillonite (MMT) is a clay material forming plate-like particles consisting of silicate layers with a thickness of 1 nm and 0.2 to 2 μm in the lateral dimension. The 2:1 layered structure constructed of octahedral sheet sandwiched between two tetrahedral sheets (see Figure 3b). Due to the specific crystal structure, MMT has high adsorption capacity along with a high net negative surface charge [52]. The hydration of the surface cations results in the poor affinity of MMT to hydrophobic substances, therefore, the surface of MMT has to be modified prior to organic PCM loading. In one of the first works devoted to PCM loading into MMT for energy storage applications, Fang and Zhang pretreated MMT with hexadecyltrimethylammonium bromide (CTAB) to load the organic PCM RT20 based on saturated hydrocarbons [53]. The loading was carried out by the blending of melted PCM with MMT particles. The loading efficiency was figured out to be 58% whereas the resulted composite had a phase change temperature of 23 °C with a latent heat of phase transition of 79.25 J/g, which were preserved after 1500 heating/cooling cycles according to the DSC analysis. Later, the same group compared the properties of three organic PCM/clay materials composed of butyl stearate, dodecanol and RT20 loaded to organically modified MMT [54]. RT20 loaded MMT demonstrated the best LHS properties and cyclic stability. The RT20/MMT composite was successfully incorporated into the gypsum wallboard and demonstrated a reduction of indoor temperature fluctuations in the test room. Sarier et al. prepared MMT loaded with *n*-hexadecane organic PCM by surfactant mediated intercalation [55]. Prior to PCM loading, MMT was treated with an anionic surfactant, a sodium salt of 4-dodecylbenzene sulfonic acid. The authors indicated the increase of efficiency of the thermal storage of PCM/MMT composite along with the loading percentage of PCM and improvement of thermal capacity and thermal conductivity of the composite comparing to bare PCM. Further, the PCM/MMT composites were prepared with lauric acid by melting intercalation [56] and stearic acid by vacuum impregnation [57]. Wang et al. compared two types of composites prepared by melting and dissolving impregnation of stearic acid into MMT [58]. Both composites demonstrated comparable loading efficiency about 47% and latent heat about 85 J/g yet the composite prepared by melting impregnation demonstrated much better thermal reliability. The recent studies were focused on more sophisticated approaches to PCM/MMT composites preparation. For instance, Peng et al. described MMT/stearic acid core/shell microcapsules prepared by self-assembly of MMT in stearic acid emulsions and compared their thermal properties with the same composites prepared by vacuum impregnation [59]. The microcapsules had larger stored latent heat (118 J/g) than that of the composites (59 J/g) that was related to a higher loading (59 vs. 35 wt%) and crystallinity of the stearic acid. Additionally, the core/shell structures demonstrated good thermal reliability with a reduction of the stored latent heat only by 8% after 100 cycles. Liao et al. prepared a PCM/MMT composite via two-stage intercalation method [60]. The first stage was ionic exchange intercalation of MMT with *n*-octadecylamine (ODA) salts followed by hydrophobic attraction of *n*-dodecane at the second stage. This approach allowed for control over the paraffin adsorption by variation of the salt amount exploited for MMT modification at the first stage, and, therefore to tune the thermal properties of the resulted composites. Xiaofeng et al. employed a multi-step preparation procedure to modify MMT with hyperbranched polyester that was further coupled with paraffin [61]. Wei and Wang described the preparation of inorganically modified MMT with HCl resulting in a gel-like MMT structure that can be loaded with paraffin PCM [62]. However, comparing to organically modified MMT loaded with the same PCM, the composite with inorganically modified MMT demonstrated almost 50% of PCM leakage and the LHS capacity two times lower. Yi et al. prepared core/shell particles by self-assembly of stearic acid latex particles and MMT nanosheets with various shell thickness [40]. The initial MMT was exfoliated by ultrasound treatment to form nanosheets and stabilized by CTAB whereas the stearic acid particles were stabilized by sodium dodecyl sulfate. They found out, that the core/shell particles with the thinnest shell demonstrated the highest loading efficiency (88%) and LHS capacity (184.88 J/g) along with the best thermal conductivity of the shell (0.46 W/mK) and the whole composite particle (0.29 W/mK). Additionally, the prepared composites demonstrated good shape stability and thermal reliability after 50 heating/cooling cycles. Further, the same group prepared 3D structures via self-assembly of MMT nanosheets and loaded them with stearic acid by vacuum impregnation [63]. The loading efficiency was up to 95% with the LHS capacity of 195 J/g. The resulted PCM/MMT composite had improved thermal conductivity (0.308 W/mK) along with the shape and thermal reliability. Wu et al. studied the controllable assembly of stearic acid within the interlayer spacing of MMT [64]. The composites were prepared by the liquid phase intercalation method. They found out that the phase transition temperature of PCM/MMT composite can be tuned by the surface charge of MMT layers affecting the intercalated interlayer amount of stearic acid, its arrangement morphology, and intermolecular interactions. Therefore, these materials can be adjusted for low-temperature storage applications.

Although PCM/MMT composites generally have better thermal conductivity than pure PCMs, some research was devoted to the preparation of PCM/MMT materials with further improved thermal conductivity by the inclusion of carbon nanostructures or metal nanoparticles. For instance, Kao et al. reported on the preparation of extended graphite (EG)/paraffin/MMT composite by molten intercalation [65]. The EG is known to have a pore structure with extended specific surface area and high thermal conductivity. The EG/paraffin/MMT composite demonstrated good cycling stability with the melting point of 41.6 °C and the latent heat of 112.21 J/g whereas the introduction of EG improved the heat transfer five-fold compared to paraffin/MMT composite without EG. 

**Figure 4:**
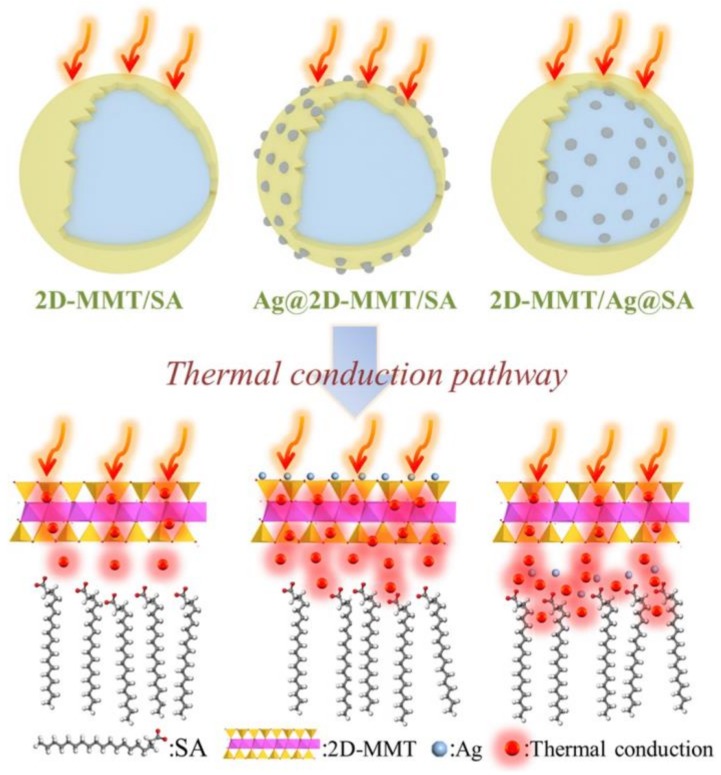
A sketch demonstrating the thermal conduction of the stearic acid/MMT core/shell composite with Ag nanoparticles embedded either into MMT shell or into the stearic acid core. Reproduced with permission from [69]. Copyright Elsevier.

Jeong et al. studied *n*-hexadecane and *n*-octadecane/MMT composites prepared with the addition of exfoliated graphite nanoplatelets [66]. Both types of composites with graphite particles demonstrated improved latent heat capacity, enthalpy of phase transition and more than 230% increased thermal conductivity comparing to the composites prepared without graphite. Tabassum et al. studied the thermal properties of the composite prepared with methyl stearate as PCM, EG, MMT, and ammonium polyphosphate [67]. In this study, the addition of EG improved the thermal conductivity of the composites by 3.5 times comparing to bare methyl stearate. Additionally, the thermal conductivity and phase change enthalpy were shown to be adjustable by the fraction of MMT and ammonium polyphosphate. Further, Li et al. described the preparation of paraffin/MMT composite with the addition of multi-walled carbon nanotubes [68]. The inclusion of carbon tubes improved intercalation and adsorption of the PCM and led to better heat transfer and thermal conductivity of the resulted composites. Zhan et al. prepared stearic acid/exfoliated MMT core/shell composites with the inclusion of Ag nanoparticles either into the core or the shell (Figure 4) in order to find out the most effective way to improve the thermal conductivity and heat transfer [69]. 

The inclusion of silver nanoparticles did not affect significantly the LHS capacity for both types of the composites (188 J/g), yet significantly improved the thermal conductivity. The addition of Ag into the shell improved thermal conductivity by 74.36% comparing to stearic acid/MMT composite while the addition to the core improved the thermal conductivity by 187.82%. Therefore, it was concluded that the PCM core was limiting the heat transfer through the capsule due to its large content. 

A summary of the thermal properties of some of the PCM/MMT composites mentioned in this section is given in Table 1.

### 3.2. Sepiolite-Based PCM Composites

**Figure 5:**
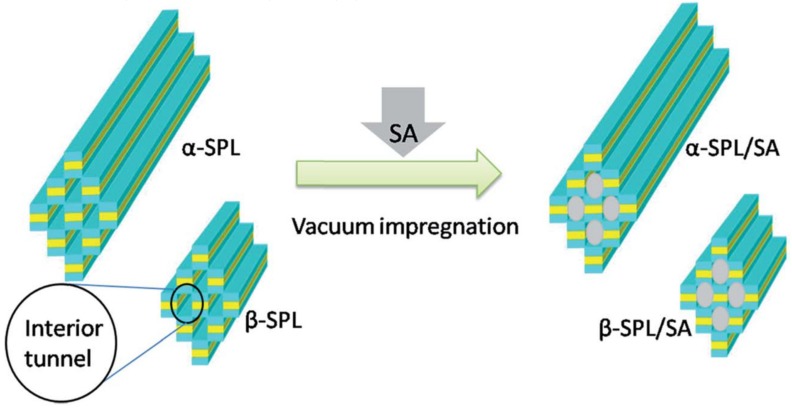
Schematic representation for preparing a-SPL/SA and b-SPL/SA composites. Reproduced with permission from [74]. Copyright The Royal Society of Chemistry.

Sepiolite is a 2:1 two-sheet type clay mineral with a fibrous structure that differs from laminar clays by having internal tunnels (see Figure 3c). The theoretical formula of ideal sepiolite crystal is Si_12_O_30_Mg_8_(OH)_4_(OH_2_)_4_·8H_2_O. The tunnels in sepiolite structure induce a fibrous morphology, which results in great adsorption capacity and rheological properties. Thus, the tunnels may contain water and other small molecules [70,71]. Sepiolite has a large specific surface area (121.605 m^2^/g) and significant porosity (with the total pore volume diameter of 3.823 cm^3^/g and an average pore diameter of 0.537 nm) [72]. The surface properties of sepiolite may be further improved by the treatment with inorganic acids. Sepiolite fibers were employed for the preparation of composites with either organic or inorganic PCM, including paraffins, fatty acids, and salt hydrates. One of the first, Wang et al. reported on the preparation dodecanol/sepiolite composite by mixing and physical adsorption of dodecanol molecules into sepiolite cavities [73]. The maximal loading efficiency of dodecanol was about 67.69 wt%. Further, Shen et al. studied the loading of stearic acid into α-sepiolite (α-SPL) and β-sepiolite (β-SPL) by vacuum impregnation [74]. α-SPL is a large bundle of fibrous crystals while β-SPL is short and thin fibrous crystals (Figure 5). The loading efficiency was found to be 60 wt% for *α*-SPL composite and 49 wt% for β-SPL. As a result, the composites had a thermal conductivity of 0.57 W/mK and 0.76 W/mK, LHS capacity of 118.7 J/g and 95.8 J/g and good thermal reliability after 200 heating/cooling cycles.

The same group studied the effect of sepiolite pretreatment on the adsorption of lauric acid and thermal properties of the resulted composites [75]. Sepiolite was treated by calcination, alkali leaching, and hydrochloric acid. The acid treatment was found to be the most effective to remove the impurities and improve the pore structure. The maximal loading capacity of acid-treated sepiolite was 60 wt%, which is 50% higher than that of raw sepiolite. The composite prepared with acid-treated sepiolite had the melting/freezing temperature of 42.5/41.3 °C with the corresponding LHS capacity of 125.2 J/g and 113.9 J/g. The measured thermal conductivity was 0.59 W/mK. Further, Konuklu et al. figured out that an ultrasound treatment had a negative effect on sepiolite due to its tubular structure while the microwave treatment promotes the preparation of the composites with improved thermal conductivity [76]. Additionally, Konuklu and Ersoy compared the thermal properties of the composites prepared by mixing paraffin or dodecanoic acid with sepiolite [77]. They found out that the paraffin had a higher adsorption ratio and can be loaded into sepiolite fibers in a more controllable way as it does not possess any functional groups like dodecanoic acid. Therefore, although the neat paraffin and dodecanoic acid have a comparable enthalpy of phase transitions (about 165 J/g), the composites loaded with paraffin had higher LHS capacity (about 65 J/g) than those loaded with dodecanoic acid (about 35 J/g). Sari et al. prepared the PCM/sepiolite composite employing capric acid/stearic acid eutectic mixture (83/17 wt%) as the PCM [78]. The form-stable composite PCM was prepared with the mass fraction of the eutectic mixture of 42 wt%. The melting temperature was 24.51/22.86 °C with the corresponding LHS capacity of 76 J/g. Additionally, composite demonstrated good thermal reliability after 1000 heating/cooling cycles. Cui et al. prepared CaCl_2_∙6H_2_O/sepiolite composite by vacuum impregnation method [79]. The sepiolite was pretreated with the hydrochloric acid, calcinated and modified with propyltrimethoxysilance. As a result, the shape stable composite loaded with 70 wt% of CaCl_2_∙6H_2_O was formed. The composition with 70 wt% of CaCl_2_∙6H_2_O was also found to be the most suitable to prevent the phase separation of the salt hydrate. Although, the PCM initially demonstrated segregation behavior, the loading to sepiolite matrix was shown to be effective to prevent the further phase separation and preserve the main phase transition peak with the highest enthalpy on DSC curves.

A summary of the thermal properties of some PCM/sepiolite composites mentioned in this section is given in Table 2.

### 3.3. Kaolinite-Based PCM Composites

**Figure 6:**
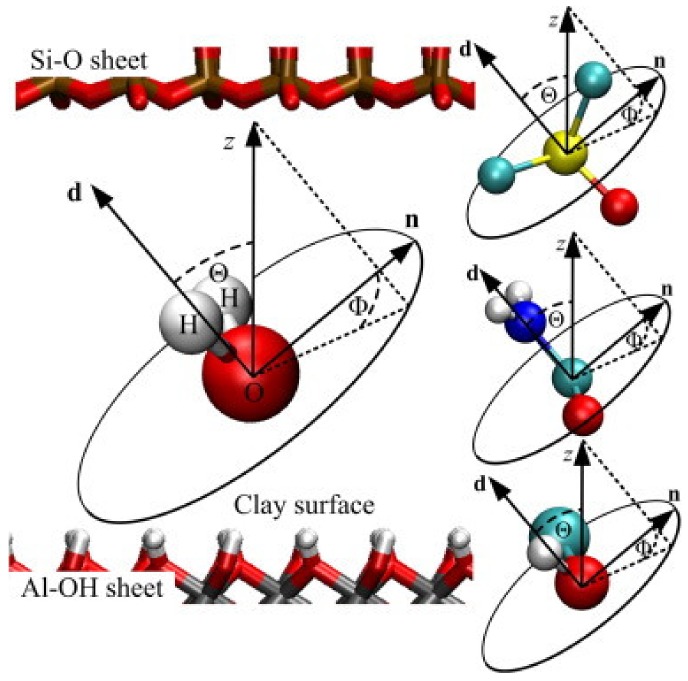
Definition of the orientation of polar molecules between kaolinite sheets. From top to bottom the graphical representations of the molecules correspond to DMSO, formamide, and methanol, respectively (–CH and –CH_3_ groups are modeled by only one site). **d** is the dipole moment of the molecule, *z* is the axis normal to the clay surface, and **n** is a normal vector of the molecular plane, except for DMSO, where **n** is normal to the plane determined by **d** and the bond vector **δ**_S__→O_. Reproduced with permission from [80]. Copyright Elsevier.

Kaolinite (KO) is the most common 1:1 two-sheet type clay mineral with the formula A1_2_Si_2_O_5_(OH)_4_. The monolayer crystal structure of kaolinite composed of one silicon-oxygen (SiO_4_) tetrahedral layer and one alumina [Al(O, OH)_6_] octahedral layer bonding together by sharing of apical oxygens (see Figure 3a). Originally, kaolinite has narrow monolayer repeat distance (0.7133 nm in ideal crystal) and low cation exchange capacity, which means that only strong polar low-molecular compounds can be intercalated into the interlayer without kaolinite pre-treatment [15,80,81]. However, the interlayered spacing can be expanded with highly polar molecules (like dimethyl sulphoxide (DMSO) or formamide) called intercalators (Figure 6) [80]. 

The other possible option is to employ polar PCM molecules. For instance, Memon et al. studied the loading of lauryl alcohol into KO by vacuum impregnation to prepare a form-stable composite [82]. Lauryl alcohol is known as one of the most polar PCM. According to DSC analysis was about the stored latent heat of 48.08 J/g, which implies the loading efficiency of 23%. The composite also had good cyclic stability with the only minor change of LHS capacity to 0.16 J/g after one month of thermal cycling tests. Liu and Young compared the thermal properties of the composites prepared by vacuum impregnation of stearic acid into KO and coal–series KO. The latter had a larger specific surface area and pore volume (according to BET), which resulted in better loading of stearic acid and, therefore, higher LHS capacity. The thermal reliability and chemical stability were better for coal–series KO composite as well. Song et al. studied the loading efficiency of lauric acid into DMSO treated KO by solution intercalation method [83]. They established that the highest loading efficiency was 48%. The samples of this composition demonstrated no PCM leakage and behaved as from stable PCM. The stored in the composite latent heat was 72.5 J/g and the thermal conductivity was measured to be 0.101 W/mK. Sari compared the thermal properties of the composites prepared by vacuum impregnation of capric acid, polyethylene glycol 600 and heptadecane into KO [84]. He found out the maximum loading efficiency of PCM forming shape stable composites was 17.5, 21 and 16.5 wt%, respectively. The corresponding latent heat calculated by DSC was 27.23, 32.80, and 34.63 J/g and the composites remained stable after 1000 heating/cooling cycles. The measured thermal conductivity the composites were relatively low (0.17, 0.18 and 0.20 W/mK), however, the addition of 5 wt% of expanded graphite improved the thermal conductivity by 35%, 50%, and 45%, respectively. Alternatively, Li et al. investigated the effect of three different forms of KO (platelet, layered, and rod) and their pore structure on the thermal storage properties of paraffin PCM [85]. The KO was pre-treated with hydrochloric acid under microwave irradiation. The PCM was loaded by vacuum impregnation with loading efficiencies of 50.9, 44.0 and 43.9 wt%, respectively. The latent heats of phase transitions were figured out to be 107.2, 94.8, and 84.1 J/g, which were, however, lower than expected values. This was attributed to the lower degree of crystallinity of the paraffin in the composites comparing to the theoretical values. Finally, the thermal conductivities were 0.67, 0.78, and 0.65 W/mK for the composites with platelet, layered, and rod KO. The same group reported on the composite prepared by loading of capric and lauric acid into DMSO treated coal-series KO [86]. The mixture of PCMs had lower phase transition temperature (16.96 °C) comparing to pure PCMs. The loading efficiency was found to be about 36.9 wt% that was corresponded to the latent heat of phase transition of 42.36 J/g. Lv et al. studied the effect of particle size and mass fraction of the kaolinite on the thermal properties of paraffin/KO composites [87]. The composites were prepared via vacuum impregnation. Comparing the composites prepared with the same ratio of PCM to KO particles (60/40 wt%) of various size, the composite with the largest particles (6.5 µm) had the highest thermal conductivity 0.413 W/mK while the latent heat was nearly the same (about 120 J/g). Comparing the composites containing a various fraction of the largest KO particles, the thermal conductivity was growing (from 0.333 to 0.547 W/mK) along with the particle fraction (from 10 to 60 wt%) and vice versa the stored latent heat was decreasing (from 175.84 to 76.66 J/g). Further, Li et al. studied the effect of KO particle size distribution on the thermal properties of the composites loaded with stearic acid [88]. The authors employed five KO particle samples with a particle size distribution from bi-modal to monodisperse. The LHS capacity of the composites was dependent on the particle size distribution due to the various loading and crystallinity of stearic acid related to the layout of the KO particles in the composite structure. The thermal conductivity was also found to be dependent on the KO particle distribution due to the establishment of an internal heat transfer network into the composites and can be tuned by the ratio between smaller and larger particles. Zuo et al. studied the effect of the interaction of stearic acid and KO on the thermal properties of the composites [89]. They found out that preliminary modification of KO with APTES prevents the formation of covalent bonds between stearic acid and AlOOH sheets that leads to the better crystallinity of impregnated stearic acid. As a result, the composite demonstrated better LHS capacity and improved cycling stability comparing to the untreated with APTES samples. Li et al. studied the effect of the additives on the thermal properties of the composite ceramics prepared with Na_2_CO_3_/K_2_CO_3_ (52/48 wt%) eutectic salt as PCM [90]. The composite ceramics was produced by the uniaxial compression method. According to their results, the addition of 10 wt% of KO to ceramics composition led to a decrease of LHS capacity by 49% compared to the sample without KO that was associated with the formation of specific microstructure between KO and eutectic salt that may affect the phase transition enthalpy eventually. On the other hand, the composites with KO demonstrated the best mass stability and prevented the leakage of the molten salt after 1440 heating/cooling cycles.

A summary of the thermal properties of some PCM/KO composites mentioned in this section is given in Table 3.

### 3.4. Diatomite-Based PCM Composites

**Figure 7:**
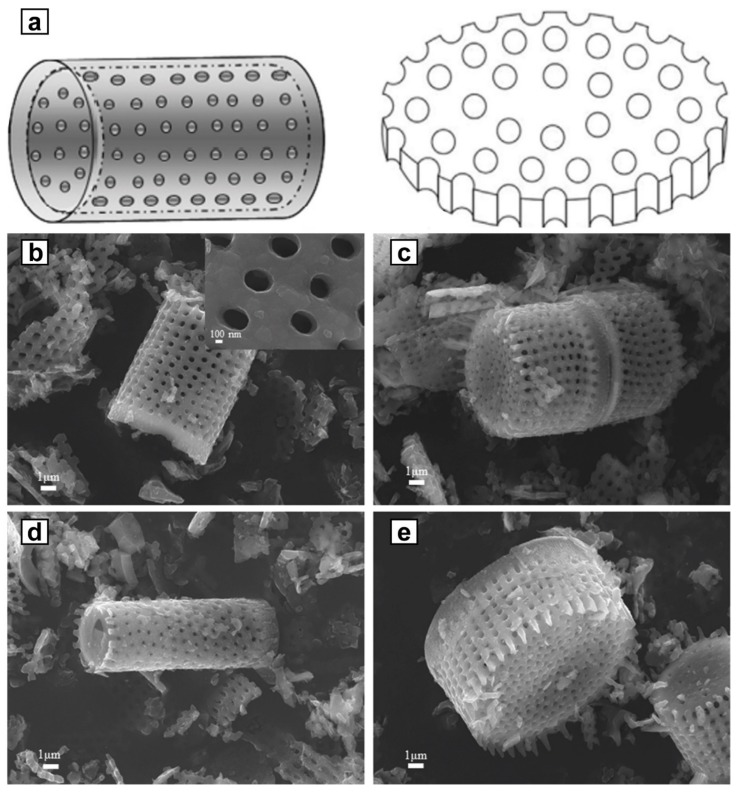
The structure diagram (**a**) and SEM images of cylindrical shape (**b**–**d**), and disc shape diatomite (**e**). Adapted with permission from [14] and [92]. Copyright Elsevier.

Diatomite is a siliceous mineral consisting of fossilized skeletal remains of diatoms unicellular algae (Figure 7). Diatomite mainly consists of silica (up to 90%), some amount of bound water (about 3–8%) and also contains alumina and the traces of other minor constituents. Diatomite has a prominent porosity (the pore radius is 0.1–3 µm and the pore volume is 0.45–0.98 cm^3^/g), a large specific surface area (33–65 m^2^/g) and a lot of hydroxyl and hydrogen bonds on the surface governing its adsorption behavior, which makes diatomite attractive support material for PCM loading [14,15,91].

To improve absorbability and purity, diatomite is typically calcinated before utilization. The calcination for 2 h at 450–500 °C allows for the effective removal of organic impurities and enhancing the porous structure [15]. The first studies of PCM/diatomite composites were devoted to loading of organic PCM like PEG [93], fatty acid esters [94], paraffins [95], and binary eutectic fatty acids mixtures [96] to diatomite particles by vacuum impregnation or fusion adsorption method.

All in all, organic PCM/diatomite composites demonstrated good thermal and chemical reliability and shape stability during the cyclic thermal test along with the suitable LHS capacity and melting temperatures for energy storage applications in buildings. Therefore, some further researches were focused on the performance of PCM/diatomite composites embedded into conventional building materials as thermo-regulating additives. For instance, Fořt et al. prepared form stable dodecanol/diatomite composite and incorporated it into cement-lime plaster [97]. The resulted composite had phase transition temperatures of 23.3 to 29.5 °C (melting) and 21.2 to 16.7 °C (freezing) with an LSH capacity of about 75 J/g without significant leakage of PCM after 100 thermal cycles. After the incorporation of the composite into the cement-lime plaster, the phase transition intervals shifted to 18.3 to 30.6 °C and 21.1 to 14.6 °C, respectively, whereas the LHS capacity reduced to 4.88 and 10.32 J/g (for the mixtures containing 8 and 16 wt% of the composite additive). Further, the same group studied the properties of plaster composition containing 8, 16, and 24 wt% of the dodecanol/diatomite composites [98]. The addition of 24 wt% to the plaster mixture improved the LHS capacity up to 15.38 J/g without a significant reduction in the mechanical performance of the plaster. The thermal properties of the composite and plaster mixture remained stable after 100 heating/cooling cycles as was revealed by DSC. Miliozzi et al. studied the thermal properties of cement mortars mixed with PCM/diatomite composite [99]. NaNO_3_/KNO_3_ (60/40 wt%) salt mixture was employed as PCM and loaded to diatomite with the ratio of salt/diatomite of 80/20 wt%. The base mortar was made of Portland cement type 42.5, sand 0–4 mm and water with the addition of 2 wt% of the PCM composite. The results of the thermal test demonstrated that the addition of a small amount of encapsulated PCM has a positive effect on volumetric thermal capacity and conductivity and also on the mechanical properties. Jin et al. loaded the eutectic mixture of stearic acid and palmitic acid to diatomite and employed the resulted composite as an additive to asphalt pavements [100]. The PCM/diatomite composite had a melting temperature of 52.93 °C and an LHS capacity of 106.7 J/g. The thermal performance tests demonstrated that the temperature-adjusting asphalt pavements included PCM/diatomite composite can reduce the peak temperature of the upper and bottom surface by up to 8.11 °C and 6.36 °C during a representative summer day. Abden et al. prepared methyl stearate/diatomite composite (with the melting temperature of 36.5 °C and LHS of 111.8 J/g) and embedded it into the foamed gypsum board [92]. The gypsum board contained about 40 wt% of the composite particles. The resulted gypsum boards were tested with respect to cooling reduction performance in a miniaturized test room outdoors. The results demonstrated that the measured peak temperature reduced by an average of 3.5 °C in the room with gypsum bards modified with PCM composite comparing to the room with the same boards without encapsulated PCM.

The other research direction is related to the preparation of novel sophisticated structures and compositions with PCM and diatomite. For instance, Guo et al. proposed to employ high-density polyethylene (HDPE) and wood fiber (WF) as the secondary encapsulated matrix to prevent the leakage of the loaded to diatomite paraffin [101]. The LHS capacity of the resulted composites can be tuned by the paraffin/diatomite ratio and the fraction of the composite particles in the polyethylene/wood fiber matrix. Zhang et al. proposed to cover the diatomite particles loaded with CaCl_2_·6H_2_O with paraffin [102]. In this formulation, every next step resulted in the reduction of the melting enthalpy from 185.6 J/g for CaCl_2_·6H_2_O to 117.1 J/g for CaCl_2_·6H_2_O/diatomite and to 108.2 J/g for CaCl_2_·6H_2_O/diatomite/paraffin composite while the melting temperature was slightly increased from 28.4 °C to 28.7 °C and 28.9 °C respectively. On the other hand, the thermal stability of the CaCl_2_·6H_2_O/diatomite composite was much better than this of pure salt hydrate and the stability of CaCl_2_·6H_2_O/diatomite/paraffin composite was increased further. Leng et al. prepared the composite PCM/diatomite material combining mixing and sintering approaches and employing NaCl:KCl (1:1.02) binary eutectic mixture as a high-temperature PCM with the melting temperature of 665 °C and melting enthalpy of 259.6 J/g [103]. Depending on the mass fraction of molten salt eutectic, the resulted composites had the melting temperature from 659 to 661 °C and melting enthalpy from 127 J/g (50 wt% of salt) to 179.3 J/g (70 wt% of salt). The sample with the 70% loading demonstrated stable phase transition temperature after 1000 thermal cycles, yet the LHS capacity reduced to 163.1 J/g due to the mass loss of about 2.2 wt%. Yang et al. reported on the preparation of hydrated salt/diatomite composites with reduced supercooling and enhanced thermal conductivity [104]. The three types of composites were prepared by mechanical impregnation of CaCl_2_·6H_2_O, CH_3_COONa·3H_2_O, and Na_2_SO_4_·10H_2_O into diatomite particles. The supercooling of the salt hydrates was significantly reduced after loading into diatomite particles and it was additionally reduced to almost 0 °C by the addition of a small weight fraction of nucleating agents. In turn, the thermal conductivity was increased by 70 to 105% compared to pure salt hydrates by the addition of 10 wt% of graphite into the composition. Konuklu et al. compared the properties of pentadecane/diatomite composites prepared by direct impregnation, vacuum impregnation, and ultrasonic-assisted impregnation methods and estimated the effect of microwave treatment of the resulted composites [76]. The composites prepared with direct impregnation of pentadecane demonstrated the highest LHS capacity and thermal conductivity with no supercooling. The microwave treatment of diatomite positively affected pentadecane adsorption increasing the LHS capacity additionally by 5 J/g. The composites prepared after diatomite microwave treatment also demonstrated higher thermal conductivity. Further, Li et al. got similar results employing a microwave-assisted acid treatment of diatomite [43]. They found out that the microwave-acid treatment results in enhanced loading capacity (by 30%) of diatomite comparing to the untreated one. Additionally, the modification of the surface and pore structure results in better crystallinity of the loaded lauric acid-stearic acid eutectic mixture. Both of these factors led to a higher LHS capacity of the composites prepared with pretreated diatomite. 

In addition, several studies were conducted for the improvement of thermal conductivity of PCM/diatomite composites by the inclusion of various carbon nanostructures like single- and multi-walled carbon nanotubes [105,106,107,108], graphite [109], graphene oxide [110], reduced graphene oxide [111], and expanded graphite [112]. The summary of the thermal properties of some PCM/MMT composites mentioned in this section is given in Table 4.

### 3.5. Halloysite-based PCM Composites

**Figure 8:**
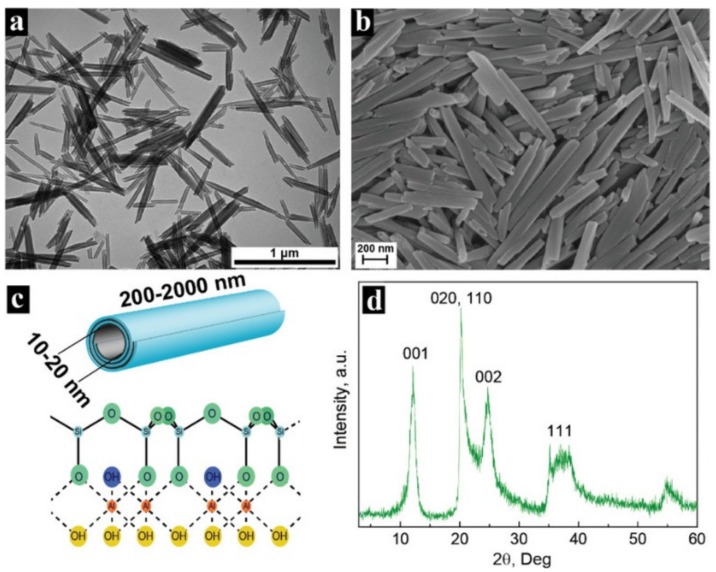
Morphology and crystal structure of halloysite (sample mined from Shanxi, China): (**a**) TEM images; (**b**) SEM images; (**c**) Crystal structure; (**d**) XRD pattern. Reproduced with permission from [117]. Copyright John Wiley and Sons.

Halloysite is a 1:1 two-sheet type clay mineral belonging to the kaolin group with the structural formula Al_2_(OH)_4_Si_2_O_5_·nH_2_O. The layer composition of halloysite is identical to those in kaolinite, however, the halloysite contains a greater amount of water in the interlayer space. The vicinity of alumina and silica layers and hydrated water results in packing disorder making the layers curl in multilayers tubular structures [113,114]. This natural tubular structure is the main feature of the halloysite that distinguishes it from the other mainly layered clay minerals. Typically, the halloysite nanotubes (HNT) have a length of 0.5–2 µm with the outer diameter varying from 50 to 200 nm and the interior lumen in the range of 10 to 50 nm [115]. It should be also mentioned that originally the halloysite is in the hydrated form with the intermediate layer of water in the roll (*n* = 2). In this case, the interlayer space is equal to 10 Å. However, the halloysite can pass the irreversible phase transition to dehydrated form (*n* = 0) when heated from 60 to 150 °C. The interlayer space of dehydrated halloysite is 7 Å [114]. Another unique feature of the halloysite related to its tubular structure is the different chemistry of positively charged Al(OH)_3_ internal surface and negatively charged SiO_2_ external surface [116]. This allows the selective modification either of the inner lumen or outer surface in order to combine the HNT with the desired agents. The representative electron microscopy images, crystal structure and XRD pattern are given in Figure 8.

To sum up, the halloysite is a natural tubular micro-container with the unique surface chemistry allowing for selective modification of lumen and outer surface. However, there are not many works related to the preparation of PCM/HNT composites to the moment. This probably might be associated with the similarity of halloysite to kaolinite that requires additional pretreatment for the intercalation of PCM materials. 

The first studies related to loading of PCM into HNT was carried out by Mei et al. They have successfully loaded stearic acid [118], capric acid [119], and paraffin [120] into the HNS lumen. The composites were prepared by mixing of PCM and HNT in ethanol solution without any pretreatment of the halloysites. The optimal ratio of the organic PCM to HNT preventing the leakage of the PCM in the molten state was found to be 60/40 wt% for all formulations. Substantially, FTIR measurements did not reveal the formation of any new bonds signifying that the composite formation is only defined by the physical interaction of the organic PCM and HNT. The DSC results demonstrated, that the LHS capacity was directly related to the loaded amount of PCM analogous to the other PCM/clay composites. Additionally, the authors have studied the effect of graphite inclusion on the thermal properties of the composites. The addition of 5 wt% of graphite to the composition did not result in any significant changes in the temperatures and enthalpies of the phase transitions, however, led to the growth of the thermal conductivity and the rates of latent heat storage and release. Further, Zhao et al. prepared the paraffine wax/HNT/graphite (45/45/10 wt%) composites by melting impregnation [121]. The resulted composite demonstrated perfect shape stability even being heated to the temperature 30 °C above the melting temperature of pure wax. The DSC analysis demonstrated good cyclic stability of the thermal properties as well. The simultaneous addition of HNT and graphite resulted in a substantial increase in the thermal conductivity of the composite up to 1.4 W/mK. Additionally, the authors proposed the concept of novel bilayer material composed of two PCM/HNT composites with different thermal conductivity with the possibility of preferential heat transfer in one direction. Liang et al. proposed to change the wettability of HNT from hydrophilic to hydrophobic by treatment with polydimethylsiloxane (PDMS) to improve the affinity of HNT to organic PCM like paraffine wax and *n*-carboxylic acids [122]. The hydrophobization of the HNT results in higher loading with organic PCM, yet the crystallinity of the loaded PCM was lower than that of pure PCM. Nevertheless, the composites prepared with PDMS treated HNT demonstrated higher LHS capacity comparing to the composites prepared with untreated HNT. Thanakkasaranee and Seo studied the thermal properties of PEG/HNT composites [123]. They found out that the compositions with a minimum of 30 wt% of HNT have significantly improved shape stability and thermal conductivity comparing to bare PEG. Conversely, the LHS capacity and melting temperature were gradually decreased along with the loaded PEG fraction. Xiang et al. proposed to improve the loading of PEG into HNT by selective acid etching [124]. The inner alumina surface of HNT can be partially dissolved by selective acid etching to improve the specific surface area and pore volume while the outer silica surface remains intact. The selective etching leaded to the pore volume and specific surface area improved from 0.243 cm^3^/g and 45.4 m^2^/g for neat HNT to 0.621 cm^3^/g and 253.4 m^2^/g for the etched HNT. By doing this, the mass fraction of loaded PEG increased from 50 to 70 wt% while the maximal LHS capacity of the resulted composite reached 112 J/g and remained stable after 100 heating/cooling cycles. 

Zhu and Shchukin reported on the inorganic PCM/HNT composite prepared by vacuum impregnation employing Na_2_HPO_4_∙12H_2_O/Na_2_SO_4_∙10H_2_O eutectic (1:1) as the inorganic PCM [125]. The 67 wt% of the eutectic mass fraction was figured out as an optimal for loading to HNT. This composition demonstrated the stable phase transition temperature and the absence of phase separation. The melting enthalpy decreased only by 7% from 142 to 132 J/g after 50 thermal cycles.

The addition of carbon nanostructures into the PCM/HNT composites significantly improves their thermal conductivity. Alternatively, the noble metal particles can be used. For instance, Song et al. synthesized the HNT decorated with Ag nanoparticles and loaded them with PEG by vacuum impregnation [126]. The mass fraction of the PEG in the composites was 45 wt%, which resulted in the latent heat of about 72 J/g. The thermal conductivity of neat PEG was determined to be 0.293 W/mK. The loading into unmodified HNT improved thermal conductivity up to 0.552 W/mK whereas the employment of Ag decorated HNT improved thermal conductivity up to 0.902 W/mK that is more than 2 times higher comparing to the pure PEG. Zhao et al. prepared the Pickering emulsion of paraffin stabilized with HNT and modified with dopamine and Ag nanoparticles to perform sun-light harvesting, light-thermal conversion and storage of thermal energy in PCM core [127]. The resulted composite had two-phase transition temperatures associated with the solid-solid (45.31 °C) and solid-liquid (59.87 °C) phase changes of encapsulated paraffin. The measured LHS capacity was 150.58 J/g indicating the capsulation efficiency of the paraffin was about 87.4%. The thermal conductivity of the composite (0.34 W/mK) was predictably higher comparing to pure paraffin (0.25 W/mK) due to the introduction of Ag nanoparticles.

Finally, several studies were devoted to the preparation of solid-solid PCM with HNT. Zhou et al. described the preparation of polyurethane-based solid-solid phase change materials employing HNT as a support material and nucleation centers increasing the rate of crystallization and the crystallinity of the confined PEG segments during the synthesis [128], which is important to improve the density of stored energy. The general synthesis approach implies the crosslinking of polyurethane using PEG as a functional moiety and hexamethylene diisocyanate biuret as a crosslinking agent. The addition of 1.02 wt% HNT to the system results in the composite with LHS capacity of 118.7 J/g, good shape stability even at the temperature over 100 °C and thermal reliability after 100 heating/cooling cycles. Further, the same group demonstrated the preparation of composite solid-solid PCM material by the crosslinking of polyurethane in the pores of HNT-graphene aerogel [129]. The resulted material contained 98.83 wt% of polyurethane. The melting temperature was 57.4 °C with the LHS capacity of 103.3 J/g, which were only slightly decreased after 100 thermal cycles.

A summary of the thermal properties of some PCM/HNT composites mentioned in this section is given in Table 5.

## 4. Conclusions and Outlook

The technology of latent heat storage is highly promising for various applications related to the accumulation and release of thermal energy. However, it is still under development at the moment. As noted recently by Shchukina et al. [20], if the sensible heat storage technologies have been widely exploited in industry and building already, the latent heat storage technologies still have to be completed to deal with the current challenges with renewable energy sources and efficient consumption of the thermal energy on a wide scale. 

In the past two decades, the studies on the phase change materials demonstrated their potential for thermal energy storage applications. However, the phase change materials hardly can be exploited in their pure form and effective encapsulation methods are required to mitigate their limitations and enhance their performance. The preparation of PCM/clay mineral composites appears as a simple and inexpensive method for PCM encapsulation. The clay containers were shown to improve the shape and thermal stability of PCMs, enhance their reliability, extend the surface area and heat transfer. An important advantage of clay containers is the clay minerals are conventional components of various construction materials so the PCM/clay composites can be used as functional additives without substantial alteration of technologies of construction material’s applications. A number of researches demonstrated that the natural clay containers after purification may be used without a preliminary treatment, which makes them environmentally friendly. However, none of the clay minerals is an ideal PCM carrier itself. The various treatment and modification approaches are generally employed to improve their properties and enhance the loading capacity.

To date, the formation of organic PCM/clay composites is the most studied and developed as the phase transition range of organic PCMs is attractive for thermal management in the civilian building. The preparation of composite PCMs with reliable shape and thermal stability is well established along with the successful elaboration of the composite PCMs in cement plasters, mortars, gypsum boards, and asphalt pavements. In turn, inorganic PCMs were considered less attractive due to the issues with phase stability and supercooling, despite their better thermal properties and lower price. However, a number of recent publications demonstrate that the formation of composite inorganic PCM/clay materials with eutectics and nucleating agents allows for avoiding phase separation and supercooling. These are exciting results as inorganic PCMs are promising for a wide range of applications including high-temperature thermal energy storage. Therefore, the further development of latent heat storage systems is closely related to inorganic PCM technologies. 

The improvement of the thermal conductivity of PCM/clay composites to enhance the rate of heat accumulation and release is another important research direction. This is the most prominent for organic PCMs. Although the inorganic clay minerals possess better thermal conductivity than organic materials, their mass fraction in the composites is generally lower than that of organic PCMs. Thus, the composite PCM/clay materials have higher thermal conductivity than neat PCMs. However, it is still relatively low and can be further improved. A number of works demonstrate various approaches to deal with this issue by the inclusion of a small mass fraction of carbon nanostructures and silver nanoparticles into the composite’s formulation.

An emerging new trend in the PCM/clay materials area is the preparation of the composites with multifunctional properties. This includes but not limited the composite materials for simultaneous control of temperature and humidity (phase change humidity control materials, PCHCMs), composite PCM/clay/Ag materials for thermal regulation with antibacterial properties, capsulation of thermal energy for catalysis and thermoelectric energy conversion.

## Figures and Tables

**Table 1:** Summary of the thermal properties of some PCM/montmorillonite composites

PCM				Preparation Method and Composition	PCM/montmorillonite Composite					Reference
PCM	LHS, J/g	T_m_/T_s_, °C	λ, W/mK		LHS, J/g	Loading, wt%	T_m_/T_s_, °C		λ, W/mK	
RT20	136.3	23.24		Blending	79.25	58	23			[53]
*n*-Hexadecane	209	17–25		Surfactant-mediated intercalation	82 93 126 98	39 45 60 47	16–30 17–39 17–28 17–31			[55]
Lauric Acid	161.56	43–44		Melting intercalation	35.2		40.7			[56]
Stearic Acid	212.8	64.5		Vacuum impregnation	118.6		63.2			[57]
Stearic Acid	177.8	60.2		Melting Impregnation Dissolving impregnation	84.48 3.6	47.5 46.9	59.9/ 55.15 8.2/ 54.6			[58]
Stearic Acid	208	54.9		Self-assembly of MTT sheets on stearic acid core Vacuum impregnation	110 59	59 35				[59]
*N*-Dodecane	243	28		two-stage intercalation method	199.7					[60]
Stearic Acid	208	-/68	0.20	Self-assembly of MTT sheets on stearic acid core	184.88 163.69 151.83	88.9 78.7 73.0	-/65.66 -/64.86 -/63.89		0.29 0.26 0.21	[40]
Stearic Acid	208	69.85/ 67.12	0.20	Vacuum impregnation into 3D MTT sheets network	198.78	95.2	69.91/ 68.07		0.31	[63]
Paraffin	128.5	41–44	0.20	Molten intercalation method with addition of extended graphite (4:36:1 for EG/paraffin/MMT)	112.21	87.78	41.6			[65]
*n*-Hexadecane *n*-Octadecane	254.7 247.6	20.84 30.4		Vacuum impregnation with the addition of 5 wt% of exfoliated graphite nanoplatelets	68.95 80.16		26.60 31.82	20.96/ 12.13 30.31/ 22.80	1.11 (0.48w/o EG) 2.12 (0.83w/o EG)	[66]
Stearic Acid	208	69.85/ 67.12	0.20	Self-assembly of MTT sheets on a stearic acid core with the inclusion of Ag NPs	188.7		90.26		0.82 (0.25 w/o Ag NPs)	[69]

**Table 2:** Summary of the thermal properties of some PCM/sepiolite composites

PCM				Preparation Method and Composition	PCM/sepiolite composite				Reference
PCM	LHS, J/g	T_m_/T_s_, °C	λ, W/mK		LHS, J/g	Loading, wt%	T_m_/T_s_, °C	λ, W/mK	
Stearic Acid	206.1	70.8/ 69.1	0.26	Vacuum impregnation into α-SPL and β-SPL fibers	118.7 (α-SPL) 95.8 (β-SPL)	6049	68.0/ 60.1 67.1/ 68.2	0.0570.76	[74]
Lauric Acid	225.4	43.2/ 41.1	0.33	Vacuum impregnation into sepiolite treated with hydrochloric acid	125.2	60	42.5/ 41.3°	0.59	[75]
Capric Acid/Stearic Acid (83/17 wt%) Eutectic	184.43	24.47/ 23.12		Blending	76.16	42	22.86/ 24.51		[78]
CaCl_2_∙6H_2_O	61	52		Vacuum impregnation, A 70/30 wt% CaCl_2_∙6H_2_O:sepiolite ratio was found to be the optimal	87.9	70			[79]

**Table 3:** Summary of the thermal properties of some PCM/kaolinite composites.

PCM				Preparation Method and Composition	PCM/kaolinite Composite				Reference
PCM	LHS, J/g	T_m_/T_s_, °C	λ, W/mK		LHS, J/g	Loading, wt%	T_m_/T_s_, °C	λ, W/mK	
Lauric Acid	161.3	40.7	0.112	Solution intercalation into DMSO-treated KO	72.5	48	47.3°/39.3°	0.101	[83]
Capric Acid	190.21	31.04		Vacuum impregnation	27.23	17.5	30.71/28.21	0.17 (0.23 with 5 wt% of EG)	[84]
Paraffin	219.1	51.51/ 53.31	0.25	Vacuum impregnation into: platelet KO Layered KO Rod KO	107.2 94.8 84.1	50.9 44.0 43.7	50.07/ 53.65 50.57/ 53.65 50.89/ 53.46	0.67 0.78 0.65	[85]
Capric Acid/ Lauric Acid (65/35%) Mixture	114.71	18.96		Vacuum into DMSO- treated KO	42.36	36.93	16.96		[86]
Stearic Acid	194.3	68.6/ 66.4	0.25	Vacuum impregnation into APTES-modified KO	118.6	63.65	68.3/ 63.7	0.4	[89]
Na_2_CO_3_/ K_2_CO_3_ (52/48%) Eutectic Salt	164.3	710.5		Uniaxial compression with the addition of 10 wt% of KO	52.98	711.6	About 1.5		[90]

**Table 4:** Summary of the thermal properties of some PCM/diatomite composites.

PCM				Preparation Method and Composition	PCM/diatomite Composite				Reference
PCM	LHS, J/g	T_m_/T_s_, °C	λ, W/mK		LHS, J/g	Loading, wt%	T_m_/T_s_, °C	λ, W/mK	
Dodecanol	170	22		Vacuum impregnation	75.8		23.3–29.5°/ 21.2–16.7		[97]
NaNO_3_/ KNO_3_ (60/40 wt%) Salt Mixture	116.0	225.7	0.7	Melting impregnation with 80/20 wt% salt/diatomiteratio	93.7		227		[99]
Stearic Acid/Palmitic Acid Eutectic	196.9	54.33		Vacuum impregnation	106.7	65.2	52.93		[100]
Methyl Stearate	217.7	36.8/ 32.7		Blending	111.8	51.3	36.5/ 33.1		[92]
NaCl:KCl (1:1.02) Eutectic	259.6	665		Mixing	127 179.3	50 70	659 661		[103]
CaCl_2_· 6H_2_O CH_3_COONa· 3H_2_O Na_2_SO_4_· 10H_2_O	195.7 270.9 249.4	28.9/ 9.0 57.4/ 26.0 31.9/ 15.4		Mechanical impregnation	123.1 168.3 155.1	65 63 63	29.1/ 23.5 57.7/ 50.0 31.7/ 24.2	0.95 (with 10 wt% of Graphite) 0.78 (with 10 wt% of Graphite) 0.85 (with 10 wt% of Graphite)	[104]
Pentadecane	174.26	31.9/ 15.4		Direct impregnation with 40/60 PCM/diatomiteRatio Vacuum impregnation with 40/6 PCM/diatomiteRatio Ultrasonic-assisted impregnation with 40/60 PCM/diatomite ratio	53.71 31.61 46.45		11.90/5.72 10.57/3.56 11.01/3.94		[76]

**Table 5:** Summary of the thermal properties of some PCM/halloysite composites.

PCM				Preparation Method and Composition	PCM/halloysite Composite				Reference
PCM	LHS, J/g	T_m_/T_s_, °C	λ, W/mK		LHS, J/g	Loading, wt%	T_m_/T_s_, °C	λ, W/mK	
Paraffin Wax	171	51–54		Melting impregnationp araffin wax/HNT/graphite (45/45/10 wt%)	68.4		52.9	1.40	[121]
Paraffin Wax Myristic Acid Palmitic Acid Stearic Acid	106.7 172.4 187.3 166	54.7 54.1 62.4 55.2		Melting impregnation into PDMS-treated HNT	44.7 72.1 58.4 70.3	62.9 45.6 51.3 58.4	52.3 48.1 46.2 52.7		[122]
PEG 35000	174.0	64.4/48.9		Melt-extrusion with PEG/HNT ratio of 90/10 wt% 80/20 wt% 70/30 wt% 60/40 wt% 50/50 wt%	152.5 138.9 115.9 103.8 96.8		64.3/47.8 63.7/47.7 63.7/47.0 63.3/45.8 63.3/45.8		[123]
Na_2_HPO_4_∙12H_2_O/ Na_2_SO_4_∙10H_2_O (1:1) Eutectic	211	38.5		Vacuum impregnation	142	67	35.8		[125]
PEG 1000	162.6	38.4/24.2	0.29	Vacuum impregnation into Ag-decorated (3 wt%) HNT	71.3	45	33.6/25.7	0.90 (0.55 w/o Ag)	[126]
